# Year in review and appreciation for 2021 reviewers

**DOI:** 10.4069/kjwhn.2021.12.13

**Published:** 2021-12-15

**Authors:** Sue Kim

**Affiliations:** Mo-Im Kim Nursing Research Institute, College of Nursing, Yonsei University, Seoul, Korea

The most significant development in 2021 for the *Korean Journal of Women Health Nursing* (KJWHN), has been the confirmation of being indexed in Scopus, with retroactive coverage from 2018 and beyond. This milestone pushes us further to continue our mission of being the dependable source for women’s health nursing, not limited to Korea but also with a wider regional and international focus.

## Year in review

This year we published a fewer number of manuscripts but have focused on some key initiatives: expanding the number of English manuscripts and hosting a series on instrument development and validity and reliability [1-4].

First, KJWHN expansion of English manuscripts has increased in comparison to 2020. In 2021, 36 out of 43 published papers (30 unsolicited, 13 commissioned manuscripts) (83.7%) and 27 out of 30 unsolicited articles (90%) were published in English. Compared to the 2020 rates of 34.2% and 21.8% respectively, this increase in English publications demonstrates our commitment to making the important contributions of our authors more visible to readers. We will continue to welcome quality English manuscripts that fit our aims and scope, especially those that contribute to women’s health nursing in the region and worldwide.

Second, our invited series [1-4] have comprehensively covered current standards and body of knowledge on how to determine psychometrics for an instrument. This series was purposively published in Korean, in order to clearly present concepts and encourage the use of standard terminology.

Another foci has been responding to the ever-decreasing birth rate in Korea, i.e., 0.84 in 2020, which is a further drop from 0.92 reported for 2019 [5]. KJWHN has continued to focus on the pressing issue of Korea’s ultra-low birth rate, with papers that have outlined strategic policy directions [6] and nursing’s role in infertility support [7], as well as expanding the roles of nurses and midwives to benefit the health of the nation [8]. Another paper illustrated examples from Japan [9], on how mobilizing nurses and midwives would serve to further gender equity, which is Goal 5 of the sustainable development goals [10]. We hope these papers will stimulate action measures at policy-level as well as individual advocacy.

Among the various topics covered in 2021, KJWHN also published two statistical reports as collaborative work with Statistics Korea and four review papers. We will continue to welcome integrative reviews, scoping reviews, and systematic reviews and/or meta-analyses that fit our aims and scope.

## Journal statistics

Data on manuscripts submitted to KJWHN this year, as of December 10, 2021, are presented in Table 1.

While continuing to strive for excellence, KJWHN will also seek ways to make the submission and review process smoother.

## Appreciation for 2021 reviewers

The developments of KJWHN were made possible thanks to the sharp eyes and tireless service of our reviewers. Warm appreciation is extended to the following reviewers:

Ahn, Suk Hee (Chungnam National University)

Bae, Kyung Eui (Dongseo University)

Chae, Hyun Ju (Joongbu University)

Chae, Myung-Ock (Cheongju University)

Cheon, Suk Hee (Sangji University)

Cho, In Sook (Inha University)

Cho, Ok Hee (Kongju National University)

Choi, Mi Young (National Evidence-based Healthcare Collaboration Agency)

Chung, Chae Weon (Seoul National University)

Chung, Mi Young (SunMoon University)

Drake, Emily E. (University of Virginia)

Ha, Ju Young (Pusan National University)

Haruna, Megumi (University of Tokyo)

Hong, Se Hoon (CHA University)

Huh, Sun (Hallym University)

Jun, Eun-Young (Daejeon University)

Jeong, Geum Hee (Hallym University)

Jo, Myung Ju (Catholic University of Pusan)

Kim, Hee Kyung (Kongju National University)

Kim, Hee Sook (Dongnam Health University)

Kim, Hyun Kyoung (Kongju National University)

Kim, Kwang Ok (Dongju College)

Kim, Kyung Won (Daegue Hanny University)

Kim, Mi Jong (Hannam University)

Kim, Mi Ok (Dankook University)

Kim, Mi Young (Woosuk University)

Kim, Moon Jeong (Pukyung National University)

Kim, Myoung Hee (Semyung University)

Kim, Su Hyun (Nambu University)

Kim, Yun Mi (Eulji University)

Ko Eun (Sunchon National University)

Lee, Ju-Young (The Catholic University of Korea)

Lee, Nae Young (Silla University)

Lee, Kyoung-Eun (Texas A & M University)

Moon, So Hyun (Chosun University)

Nho, Ju Hee (Jeonbuk National University)

Park, Mee Ra (Changshin University)

Park, Mi Jeong (Hoseo University)

Park, Seo A (Gyeongbuk College of Health)

Park, So Mi (Yonsei University)

Shimpuku, Yoko (University of Hiroshima)

Shin, Gi Soo (Chung-Ang University)

Shorey, Shefaly (National University of Singapore)

Song, Ju Eun (Ajou University)

Sung, Mi Hae (Inje University)

Special congratulations to Professors Kyung Eui Bae (Dongseo University), Hyun Ju Chae (Joongbu University), and Gi Soo Shin (Chung-Ang University), who were selected as "Best Reviewer 2021."

## Figures and Tables

**Table 1.** Basic statistics on manuscripts submitted to the Korean Journal of Women Health Nursing in 2021

Category	Data	Notes
Commissioned manuscripts (n)	13	5 Editorials, 4 Issues & Perspectives, 4 Invited Papers
Unsolicited manuscripts (n)	48	
Accepted manuscripts (n)	30	
Non-accepted manuscripts (n)	11	8 Rejected, 2 rejected before review, 1 withdrawn
Manuscripts reviewed and determined (n)	38	30 Accepted and published, 8 rejected
Manuscripts under review or revision (n)	9	
Acceptance rate (%)	63.8	30/47=0.638
Median time from submission to acceptance (days)	53	
